# Genome-wide CNV investigation suggests a role for cadherin, Wnt, and p53 pathways in primary open-angle glaucoma

**DOI:** 10.1186/s12864-021-07846-1

**Published:** 2021-08-04

**Authors:** Valeria Lo Faro, Jacoline B. ten Brink, Harold Snieder, Nomdo M. Jansonius, Arthur A. Bergen

**Affiliations:** 1 Department of Ophthalmology, University of Groningen, University Medical Center Groningen, Groningen, The Netherlands; 2 Departments of Clinical Genetics and Ophthalmology, Amsterdam University Medical Center (AMC), Location AMC K2-217 | AMC-UvA, P.O.Box 22700, 1100 DE Amsterdam, The Netherlands; 3 Department of Epidemiology, University of Groningen, University Medical Center Groningen, Groningen, The Netherlands; 4 Department of Ophthalmology, Amsterdam UMC, Location AMC, Amsterdam, The Netherlands; 5 Netherlands Institute for Neuroscience (NIN-KNAW), Amsterdam, The Netherlands

**Keywords:** Primary open-angle glaucoma, CNV, WNT signalling, p53, Cell adhesion

## Abstract

**Background:**

To investigate whether copy number variations (CNVs) are implicated in molecular mechanisms underlying primary open-angle glaucoma (POAG), we used genotype data of POAG individuals and healthy controls from two case-control studies, AGS (*n* = 278) and GLGS-UGLI (*n* = 1292). PennCNV, QuantiSNP, and cnvPartition programs were used to detect CNV. Stringent quality controls at both sample and marker levels were applied. The identified CNVs were intersected in CNV region (CNVR). After, we performed burden analysis, CNV-genome-wide association analysis, gene set overrepresentation and pathway analysis. In addition, in human eye tissues we assessed the expression of the genes lying within significant CNVRs.

**Results:**

We reported a statistically significant greater burden of CNVs in POAG cases compared to controls (*p*-value = 0,007). In common between the two cohorts, CNV-association analysis identified statistically significant CNVRs associated with POAG that span 11 genes (*APC*, *BRCA2, COL3A1, HLA-DRB1, HLA-DRB5, HLA-DRB6, MFSD8*, *NIPBL, SCN1A, SDHB*, and *ZDHHC11*). Functional annotation and pathway analysis suggested the involvement of cadherin, Wnt signalling, and p53 pathways.

**Conclusions:**

Our data suggest that CNVs may have a role in the susceptibility of POAG and they can reveal more information on the mechanism behind this disease. Additional genetic and functional studies are warranted to ascertain the contribution of CNVs in POAG.

**Supplementary Information:**

The online version contains supplementary material available at 10.1186/s12864-021-07846-1.

## Introduction

Glaucoma is a common and chronic eye disease that damages the optic nerve (ON), and is one of the main causes of irreversible blindness in the world. Primary open-angle glaucoma (POAG) represents the most prevalent type of this disease. Its distribution varies between populations, ranging from 1 to 4% in Europe and from 2 to 7% in African countries [1, 2]. POAG is clinically characterized by progressive excavation of the optic disc, retinal ganglion cells (RGCs) degeneration and visual field deficit [3]. Risk factors that contribute to POAG are increased age, increased intraocular pressure (IOP), positive family history of glaucoma, as well as having African ancestry and/or myopia [4]. POAG is a genetically complex disease, and many genetic factors have been identified that play a role in its pathogenesis [5, 6].

Previous linkage and subsequent mutation analysis in families have found several candidate genes implicated in POAG. Some of these genes are cytochrome P450 family 1 subfamily B polypeptide 1 (*CYP1B1*), myocilin (*MYOC*), optineurin (*OPTN*), and TANK-binding kinase 1 (*TBK1*), WD repeat domain 36 (*WDR36*) [7]. In addition, genome-wide association studies (GWASs) have successfully identified and confirmed associations in at least 127 loci [8]. These loci span genetic variation in a number of likely candidate genes, including *CDKN2B-AS1*, *TMCO1*, *CAV1/CAV2*, *SIX1/SIX6*, *GAS7*, *ARHGEF12*, *TGFBR3*, *TXNRD3*, *ATXN2*, *FOXC1*, and *C12ORF23* [9–15]. However, even if taken together, the genetic common variants identified in these genes only explain a small proportion of the genetic contribution to the disease [16].

In 2004, with the advent of microarray technologies, a new type of rearrangements in the DNA, called copy number variation (CNV), was discovered [17, 18]. A CNV represents a genomic rearrangement that, compared to the normal diploid genome reference, varies in terms of number (deletions and duplications), and it can involve a region of lengths ranging between 1 kilobases (Kb) and many megabases (Mb). This type of rearrangement is distributed in the whole genome, representing an important source of genetic variation (around 12%) [18–21]. CNVs can encompass many genes or regulatory sequences and they exert their influence by modifying gene expression [22]. The mechanism that underlies this process can occur through translocations, inversions, or by interacting with regulatory elements [23]. For example, a CNV resulting in a DNA deletion causes a reduction of the gene dosage compared to the normal expression or it can mask the expression of a recessive or pathological allele. A CNV in duplication can instead lead to overexpression of relevant genes, or cause gene dosage alterations by disrupting their integrity or their regulatory elements, causing gene dosage alterations [24]. Redon et al. introduced the concept of CNV-Regions (CNVRs), a combination of overlapping CNVs in different subjects. Compared to a single CNV, a CNVR can result in a much larger effect through an alteration in the same pathway [23].

Techniques to detect CNV are the comparative genomic hybridization (CGH) arrays, SNP arrays, and Next-Generation Sequencing (NGS). All aforementioned techniques produce large-scale data that needs algorithms and software to be efficiently analysed. SNP array genotyping offers a number of advantages compared to other techniques, such as CGH arrays and NGS. Some of these are the high genomic coverage, the high throughput and reliability, as well as the relatively low costs. Therefore, many studies are based on SNP array data to analyse CNV [25].

Thanks to the improvement of genome-wide maps and detection techniques, it has become clear that CNVs are involved in many complex genetic traits and diseases [26]. Indeed, CNVs have been associated with human complex traits such as susceptibility to HIV infection, birth defects, autism, intellectual disabilities, type 1 diabetes, schizophrenia and rheumatoid arthritis [27–32]. Nevertheless, for POAG, CNV detection is still quite an unbeaten path [33]. Three previous studies suggested that CNVs spanning the *DMXL1*, *PAK7*,*TBK1*, *TULP3*, *PAX2*, and *GALC* genes contribute to the genetics of POAG [34–36]. However, since the eye is known to be sensitive to the effect of gene dosage, it is probable that there are potentially more CNVs and related genes that contribute to the susceptibility of this phenotype [37–40]. The discovery of new associations between CNVs and POAG, may lead to a better understanding of the aetiology underlying POAG, and also of its genetic predisposition.

In order to explore the role of CNVs in POAG, we investigated whether CNVRs can help to detect genes and mechanisms that influence the susceptibility to POAG. To this end, we selectively identified high quality CNVRs by analysing two independent cohorts and by combining results from each study using different bioinformatics tools with the purpose to perform a CNV burden test, and a case-control association. Next, we further investigated the potential functions of the genes spanned in CNVRs in a gene set overrepresentation analysis, and after combining the results, in a gene pathway analysis. We also took in consideration the expression in ocular tissues (optic nerve head, optic nerve, retina, and trabecular meshwork) of all the genes contained in the significant CNVRs.

## Materials and methods

### Study population

The study was conducted in two independent datasets, both comprising glaucoma patients and controls. First, we used DNA samples from the Amsterdam Glaucoma Study (AGS) discovery cohort consisting of POAG patients (*n* = 141) and control subjects (*n* = 137). This cohort is a hospital-based, genetically mixed population from an urban area [41]. The second cohort consisted of Dutch individuals with POAG (*n* = 612) from the hospital-based Groningen Longitudinal Glaucoma Study (GLGS) study and control subjects (*n* = 655) from the population-based University Medical Center Groningen (UMCG) Genetics Lifelines Initiative (UGLI), addressed in this study as GLGS-UGLI cohort. The participants of the AGS and GLGS-UGLI are predominantly of Caucasian origin. The demographic characteristics of the two cohorts are reported in Table 1.

**Table 1:** Characteristics of the AGS and GLGS- UGLI cohorts reported as median, interquartile range (IQR), and percentage

	AGS		GLGS-UGLI	
	*Cases (n = 141)*	*Controls (n = 137)*	*Cases (n = 637)*	*Controls (n = 655)*
Age (median [IQR])	70 [66.0. 76.0]	74 [64.5. 79.0]	73 [66.0. 80.0]	68 (65.0 .72.0]
Gender. female. n (%)	65 (43.9)	82 (59.8)	282 (44.2)	282 (43)

The study followed the tenets of the Declaration of Helsinki and was approved by the ethics boards of the University Medical Center of Amsterdam (UMC) and University Medical Center Groningen (UMCG) (2013–327). All participants provided written informed consent.

In AGS, all individuals underwent ophthalmoscopy and biomicroscopy with a 90 dioptres lens, and digital stereo images of the optic nerve head (ONH) were taken after mydriatic drops. POAG cases had to have glaucomatous optic neuropathy vertical cup-disc ratio (VCDR) > 0.7 with corresponding glaucomatous visual field loss in at least one eye or a VCDR ≥0.8 when no visual field was available. Control subjects from the AGS cohort were selected from age-similar health, and not closely related individuals to avoid the risk of false positives, aged 60 years or older with a VCDR ≤0.6 on ophthalmoscopy and fundus photography, and without eye abnormalities.

For the GLGS-UGLI, patients were selected from the GLGS database of the Ophthalmology department of the UMCG. Cohort characteristics are described in more detail by Heeg et al. (2005) [42]. In those participants who visited the department of Ophthalmology in 2015, and were classified as having POAG, we collected blood samples for genetic research in glaucoma. For POAG, we required a reproducibly abnormal visual field defect in at least one eye, compatible with glaucoma and without any other explanation. We further required the presence of glaucomatous optic neuropathy with VCDR ≥0.8, an open angle on gonioscopy, and no signs of pigment dispersion, pseudoexfoliation, or secondary glaucoma. Control subjects from the UGLI cohort have been selected using a proxy definition, and were selected from healthy, and not closely related individuals within the UGLI and the GLGS cohorts [43].

The investigated cohorts are both Dutch but there are a number of potential differences. Even though the Netherlands is a small country, principal component analysis has already detected the presence of a genetic country gradient (e.g. with an increased rate of homozygosity in the northern provinces), indicating that the southern people are more genetically heterogeneous than the northern individuals [44, 45]. This has been shown to lead to some phenotypic differences, for example, for height and eye colour traits [45, 46]. The AGS cohort is a hospital-based population from the urban area of the capital of Amsterdam. This makes it possible that the genetic make-up of these individuals is more heterogeneous compared to individuals of the GLGS-UGLI cohort who are from a more rural area of the Northern provinces.

### Genotyping and SNP- sample- quality controls

Genomic DNA was extracted from the peripheral blood, using Gentra Systems Purogene chemistry and 100 ng of DNA was loaded on an agarose gel for checking the integrity. For all cohorts the genotyping was done using the Illumina Infinium Global Screening Array® (GSA) MultiEthnic Disease beadchip version, which contains 692,367 markers. Genotyping data was analysed using Illumina’s GenomeStudio software v.2.0 (Illumina). The signal intensities for each SNP calculated by GenomeStudio, were normalized using an Illumina Custom algorithm to generate LogR ratios (LRR) and B Allele Frequencies (BAF) for the CNV detection. The LRR represents a normalized measurement of the intensity at each SNP and in diploid autosomal regions the LRR value is approximately zero; LRRs lower than zero may indicate a deletion, LRRs higher than zero a duplication. BAF represents the contribution of the allele B to the total copy number and its value ranges from 0 to 1. BAFs close to 1 indicate homozygosity for the B alleles; BAFs close to 0 indicate homozygosity for the A alleles. Values close to 0.5 indicate a heterozygous genotype. In the current study, the values of LRR were used to assess the changes in copy number. Sex chromosomes and mitochondrial SNPs were removed from this analysis based on their high false-positive rates [47, 48].

Bioinformatics based quality control was performed on SNP and sample level. Specifically, for SNP the minimal call rate was set to 99% and variants showing deviation from Hardy-Weinberg equilibrium (*p* < 1 × 10–6) were excluded from further analysis. Samples were removed if there was: a gender mismatch, > 5% missingness in the genotype data, excess heterozygosity, a potential family relation (based on an estimate of proportion of alleles shared identical by descent; π > 0.1875), or non-European ancestry (according to a multidimensional scaling analysis conducted using data from the phase 3 of the 1000 Genomes Project) [49]. These analyses were conducted using PLINK v1.9 [50].

### Detection and quality control of copy number variants

**Fig. 1:**
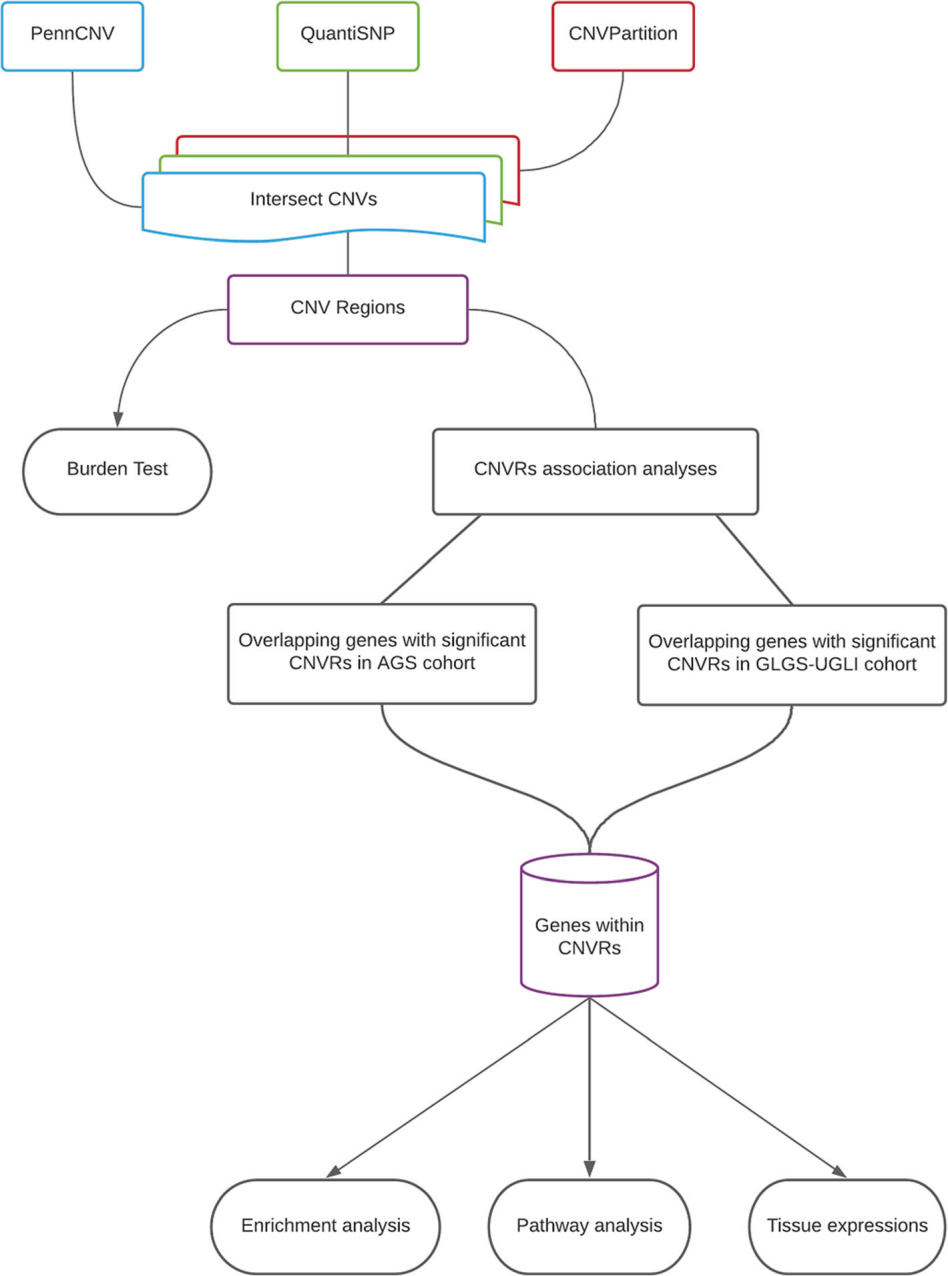
Study design

The workflow of this study is described in Fig. 1.

Detection of the autosomal regions for CNVs was done using three different programs: PennCNV v.1.0.5, QuantiSNP 2.0 and the GenomeStudio plug-in cnvPartition 3.2.0 (Illumina Inc., USA) [51, 52]. Criteria for the exclusion of potential low quality CNV data were chosen for each algorithm as described in detail below.

CNVs were called on samples using PennCNV v.1.0.5 on raw signal intensity data and the libraries provided with the software. PennCNV implements a hidden Markov model that integrates multiple sources of information to infer CNV calls [51]. PennCNV software combines different parameters, such as LRR and BAF at each SNP marker, distance between neighbouring SNPs and allele frequency of SNPs. Population Frequency of B Allele was calculated specifically for this analysis and calling was performed using the detect_cnv.pl script provided within the program. A stringent quality control on sample level was applied employing exclusions of the standard deviation of Log R Ratio (SD of LRR) > 0.35, BAF drift > 0.01 and GC-content fluctuation of signal intensity > 0.05. These thresholds were established based on the strict quality parameter values specified by PennCNV. Samples were dropped from the analysis if they had > 50 CNV calls in order to further reduce the number of potential false positives. On the CNV level, CNVs were excluded if they counted less than five probes and/or less than 5 kb in length, and CNVs with confidence scores less than ten were also removed from further analysis. Finally, CNVs were removed if at least 50% of the CNV call overlapped with telomeric, centromeric or immunoglobulin regions or with segmental duplications, since segmental duplications show highly repetitive loci and lead to excessive spurious calls. The “clean_cnv.pl” script was used to merge CNVs separated by a gap less than half of their combined length.

The second program used for CNV detection was QuantiSNP 2.0, which is based on an Objective Bayes Hidden-Markov Model for CNV analysis in Illumina Infinium SNP genotyping data. This program sets an a priori probability of observing copy number changes and the hidden state represents the unknown copy number at each SNP site. When a copy number variation is detected, QuantiSNP assigns a Bayes Factor to all of the possible combinations of copy variants. A GC content-based correction for Build 37 was applied on the data to avoid “genomic waves” [53]. After CNV detection, samples with SD of LRR > 0.25, SD of BAF > 0.3, outlier rate > 0.01, and with more than > 50 CNV calls were excluded. These thresholds were established based on the quality parameters values specified by previous CNV studies [54, 55]. The list of CNVs was obtained applying a minimum probe count of five, length greater than 5 kb, and considering the highest values of Log Bayes Factor (MaxLogBF) with a minimum threshold major than ten. The highest value provides more evidence for the existence of the CNVs.

The third program used for CNV detection was cnvPartition. It is based on the assumption that the majority of CNVs in the human genome vary between 0 and 4 copies. It assigns the copy genotypes modelling the LRR and BAF as simple bivariate Gaussian distributions. The program was run by setting a confidence threshold of 35, GC wave-adjustment, and set a minimum probe count of 5. Finally, CNVs with a length less than 5 kb were excluded.

Only CNVs meeting all strict quality criteria set for each program, detected by at least two algorithms, reciprocally overlapping, and with the type of copy number change consistent, were merged and included in subsequent analyses as high-quality CNVRs. Intersection of PennCNV, QuantiSNP and cnvPartition results was performed using the command “intersect” of BEDOPS v2.4.39 [56]. Only CNVRs present in at least 1% of individuals in each cohort were kept for further analysis. The CNVRs were defined by the innermost boundaries of the overlapping segments. All the data presented were generated in NCBI Build 37/UCSC hg19. Graphic representations of the distribution of CNVRs in the whole genome were generated using the R package karyoploteR [57].

### Power calculations

Power analyses for detection of CNVs in a total of 278 samples in the AGS cohort and 1292 samples in the GLGS-UGLI cohort in a χ^2^ test Goodness-of-fit test, indicates we have a 30% power and 90% power, respectively, to detect a small effect (Cohen’s w = 0.1). Power calculations were performed with G*Power v3.1.9.4 [58].

### Copy number variants burden test

The program PLINK v.1.07 was used to perform global burden analysis for common CNVs between POAG cases and controls in both of our studies. This approach analyses CNVs and examines whether the cases show a greater CNV burden compared to controls using permutation test 1-sided (*n* = 10,000). We evaluated the average number of CNVs and the proportion of samples with one or more CNVs. A gene list file (glist-hg19) was supplied to test the number of genes spanned by CNVs, their number with at least one gene and the number of genes per total kb. Since a CNV can possibly interrupt functional gene sequence, we considered a gene as a functional unit and we tested the hypothesis that any type of CNV affecting the normal diploid reference of the gene was associated with POAG. The human genes positions (UCSC, hg19) were obtained from the UCSC Genome Browser website [59]. In this analysis, *p*-values < 0.05 were considered significant.

### Copy number variants association test

Independently for each study, the frequency of overlapping CNVRs in cases and controls were compared in an association analysis using two-tailed Fisher’s exact tests. We only considered regions spanning genes and outside of segmental duplications. A *p*-value less than 0.05 was considered nominally significant. False Discovery Rate (FDR) multiple testing correction was calculated. The analysis was conducted using the R package CoNVaQ for CNV-based association studies between two groups [60].

### Functional annotation, gene set overrepresentation and pathways analysis

Functional classification and gene set overrepresentation analysis were conducted for candidate genes in the statistically significant associated CNVRs. The list of genes produced was used to test the over or under representation of the gene set using a Fisher test with Bonferroni correction in PANTHER (Protein ANalysis THrough Evolutionary Relationships) available at http://www.pantherdb.org/ [61, 62]. This test compares a test gene list to a reference gene list of *Homo sapiens* provided by the program, in order to calculate if a particular class (e.g. molecular function, biological process, cellular component, PANTHER protein class, the PANTHER pathway or Reactome pathway) of genes is overrepresented or underrepresented [63]. In addition, we evaluated whether pathway classification analysis in PANTHER could help to prioritize the biological pathways most likely to be involved in the disease aetiology.

Finally, we performed a CNV enrichment analysis implemented in PLINK using its -cnv-enrichment-test, with permutation (*n* = 10,000) on all CNVs, in order to identify if CNVs identified in cases were enriched for specific pathways compared to controls [64]. Specifically, the set of genes overlapping CNVs is compared to the genes belonging to a pathway, using a two-tailed Fisher’s test. A strength of the --cnv-enrichment-test is that it controls for differences in size and rate distribution of genes and CNVs.

### Expression of associated copy number variants in the ocular tissue database

The Ocular Tissue Database (OTDB) was questioned to evaluate the expression levels of the genes identified in the CNVR genome wide-scan to further evaluate their potential involvement in POAG [65]. Briefly, this database contains microarray expression data of microscopically dissected post-mortem material of ten human eye tissues. The data reported in this database was analysed using the Affymetrix Probe Logarithmic Intensity Error Estimation (PLIER) package in order to obtain expression values for each gene. The levels of gene expression are indicated by PLIER normalized values. These numbers are calculated by GC-background correction, PLIER normalization, log transformation and z-score calculation. We probed the expression of associated genes in tissues relevant to POAG, such as optic nerve head (ONH), optic nerve (ON), trabecular meshwork (TM) and retina since these are the most affected tissues in glaucomatous eyes. Specifically, we evaluated which genes from our gene-list, composed of 16 genes identified in the AGS in the association analysis and 76 genes in the GLGS-UGLI, were very high and very low expressed in ON, ONH, TM, and retina. For this purpose, we ranked the OTDB-derived expression of these genes in percentiles. Then we took in consideration the genes in our list with an expression level higher than the 90th or lower than 10th percentile.

## Results

### Sample status and quality control

Characteristics of the AGS and GLGS-UGLI cohorts are reported in Table 1. After the quality control conducted separately for each study, in the AGS cohort a total of 26 out of the 278 individuals were excluded from the analysis due to relatedness, insufficient call rate, poor quality due to elevated SD of LRR and BAF, and excessive number CNVs. The quality procedures left a total of 131 individuals with POAG and 121 controls. No significant difference in age between POAG cases and controls was observed. In the GLGS-UGLI, the POAG cases and control subjects (controls selected using a glaucoma proxy definition) were matched for age and gender in a 1:1 ratio, using the R package MatchIt with nearest-neighbor matching [43, 66]. The same quality control procedures were applied in this cohort, in which 53 individuals were excluded leaving 584 cases and 655 controls for further analysis. No significant difference of gender and age between POAG cases and controls was observed.

### Copy number variants detection and burden

The detection of CNVs was performed using PennCNV, QuantiSNP and cnvPartition. For methodological details, see the Subject and Methods section. In the AGS cohort, CNVs that passed the filters were 1791 CNVs in cases and 1466 CNVs in controls. More specifically, 1187 deletions occurred in the cases and 912 in the controls, while 604 duplications were observed in the cases and 554 in the controls. Overall, 100% of CNVs overlapped partially or in total with at least one gene in POAG cases and with 98% in controls. A significant difference in the CNV burden analysis was observed in the rate of CNVs between cases and controls (*p*-value = 0.007), meaning that a highly significant greater CNV burden was present in individuals with POAG compared to the controls. In the GLGS-UGLI cohort, after all quality controls 584 cases and 655 controls passed the filters, with respectively 10,031 and 7148 CNVs remaining for further analysis. A total number of 6506 deletions occurred in the cases and 2554 in the controls. Conversely, 3525 duplications were observed in the cases and 4594 in the controls. In cases, all the CNVs were found in partial or complete overlap with at least one gene, whilst in controls the overlap was 99%. Including all the deletions and duplications identified, the CNV burden analysis showed a significant higher burden in the difference in the cases versus controls (*p*-value = 9.99 × 10^− 5^), confirming the same trend observed in the AGS cohort.

### Copy number variants-based association analysis

**Fig. 2:**
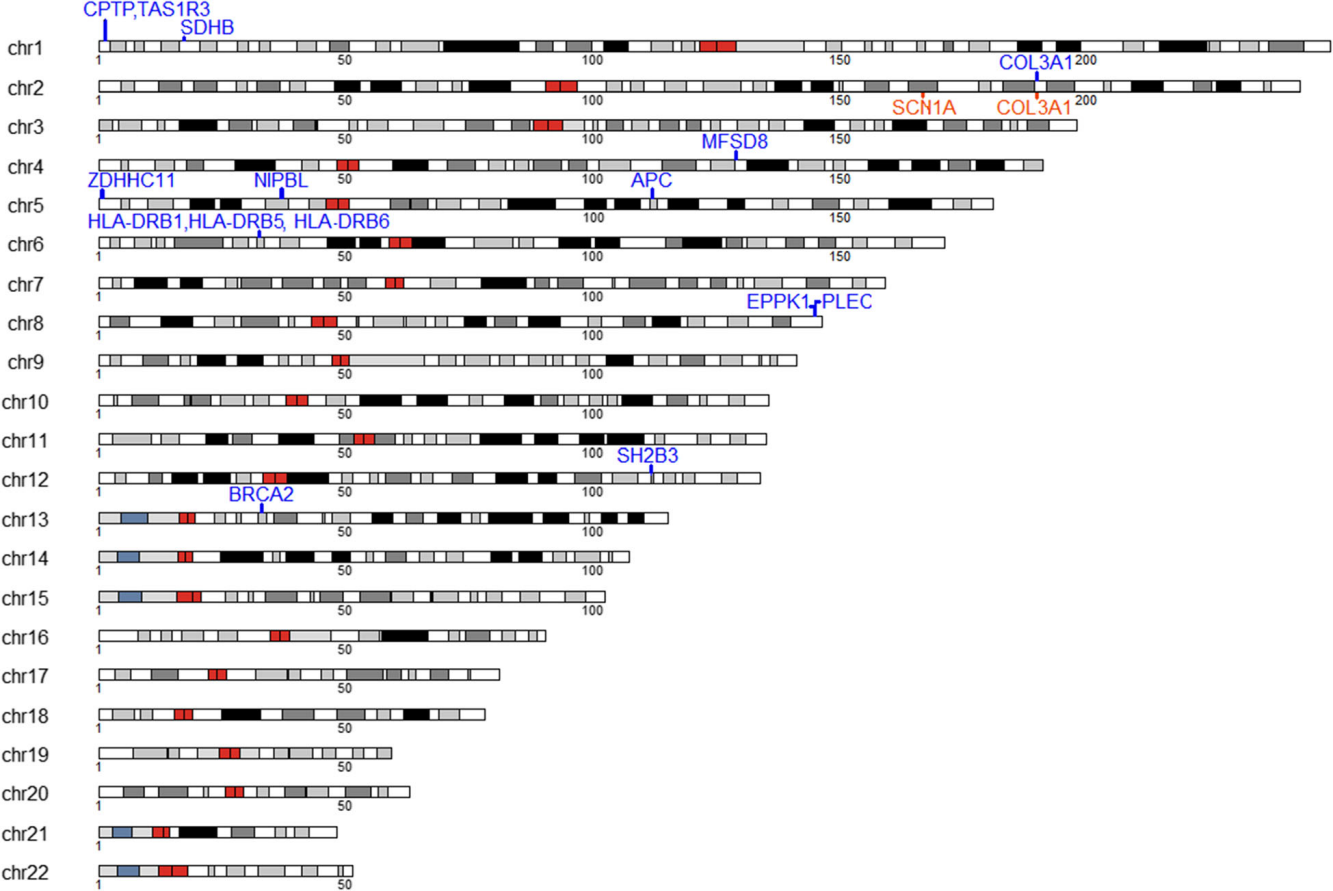
Karyoplot of the distribution of the CNVRs in the AGS cohort. Blue- and orange-coloured bars represent loss and gain events, respectively

In the AGS cohort we conducted a CNVR-based association study between POAG cases and controls. Sixteen of all identified CNVRs reached the nominal level of statistical significance (*p*-value less than 0.05) and in 14 out of 16, the CNVRs were more frequent in POAG cases compared to controls. After applying FDR correction (FDR = 5%), there were six significant associations in the *BRCA2, EPPK1, MFSD8, PLEC, SDHB*, and *SH2B3* genes*.* The CNVRs spanning with the genes, *MFSD8* and *BRCA2* reached the highest level of significance (respectively, with *p*-value = 0.0005 and *p*-value = 0.0001). The CNVR containing *MFSD8* was a deletion and was found in 13% of cases and 1.65% of controls. The CNVR overlapping with *BRCA2* was a deletion and reported in 37.4% of cases and 15.7% of controls. The results and a graphic representation of the CNVRs detected in this analysis are reported in Table 2 and Fig. 2.

**Table 2:** Significant CNVRs identified in the AGS cohort

Chromosome	Start (bp)	End (bp)	Length (bp)	Type	***P***-value	Adjusted ***p***-value	***Gene***	Cases %	Controls %
1	1,262,701	1,270,132	7433	Loss	7.09e-03	1.63e-01	*CPTP, TAS1R3*	16	4.96
1	17,361,781	17,371,377	9598	Loss	6.22e-04	2.56e-02	*SDHB*	14.5	2.48
2	166,854,547	166,866,353	11,808	Gain	3.02e-02	4.65e-01	*SCN1A*	0.763	5.79
2	189,850,694	189,871,691	20,999	Gain	1.15e-02	2.10e-01	*COL3A1*	0	4.96
2	189,850,694	189,871,691	20,999	Loss	3.71e-02	4.65e-01	*COL3A1*	6.11	0.826
4	128,851,902	128,859,998	8098	Loss	5.54e-04	2.56e-02	*MFSD8*	13	1.65
5	700,560	840,586	140,028	Loss	3.71e-02	4.65e-01	*ZDHHC11*	6.11	0.826
5	37,000,942	37,010,310	9370	Loss	7.29e-03	1.63e-01	*NIPBL*	6.11	0
5	37,024,691	37,046,283	21,594	Loss	3.71e-02	4.65e-01	*NIPBL*	6.11	0.826
5	112,102,584	112,111,355	8773	Loss	5.85e-03	1.63e-01	*APC*	13	3.31
6	32,496,275	32,520,907	24,634	Loss	3.68e-02	4.65e-01	*HLA-DRB1,HLA-DRB5, HLA-DRB6*	29	17.4
6	32,537,353	32,561,576	24,225	Loss	8.61e-03	1.73e-01	*HLA-DRB1,HLA-DRB5, HLA-DRB6*	13.7	4.13
8	144,943,389	144,952,259	8872	Loss	6.36e-04	2.56e-02	*EPPK1*	26.7	9.92
8	144,994,028	145,000,413	6387	Loss	6.29e-04	2.56e-02	*PLEC*	24.4	8.26
12	111,856,187	111,885,143	28,958	Loss	1.15e-03	3.87e-02	*SH2B3*	47.3	27.3
13	32,912,236	32,921,033	8799	Loss	1.15e-04	2.30e-02	*BRCA2*	37.4	15.7

**Fig. 3:**
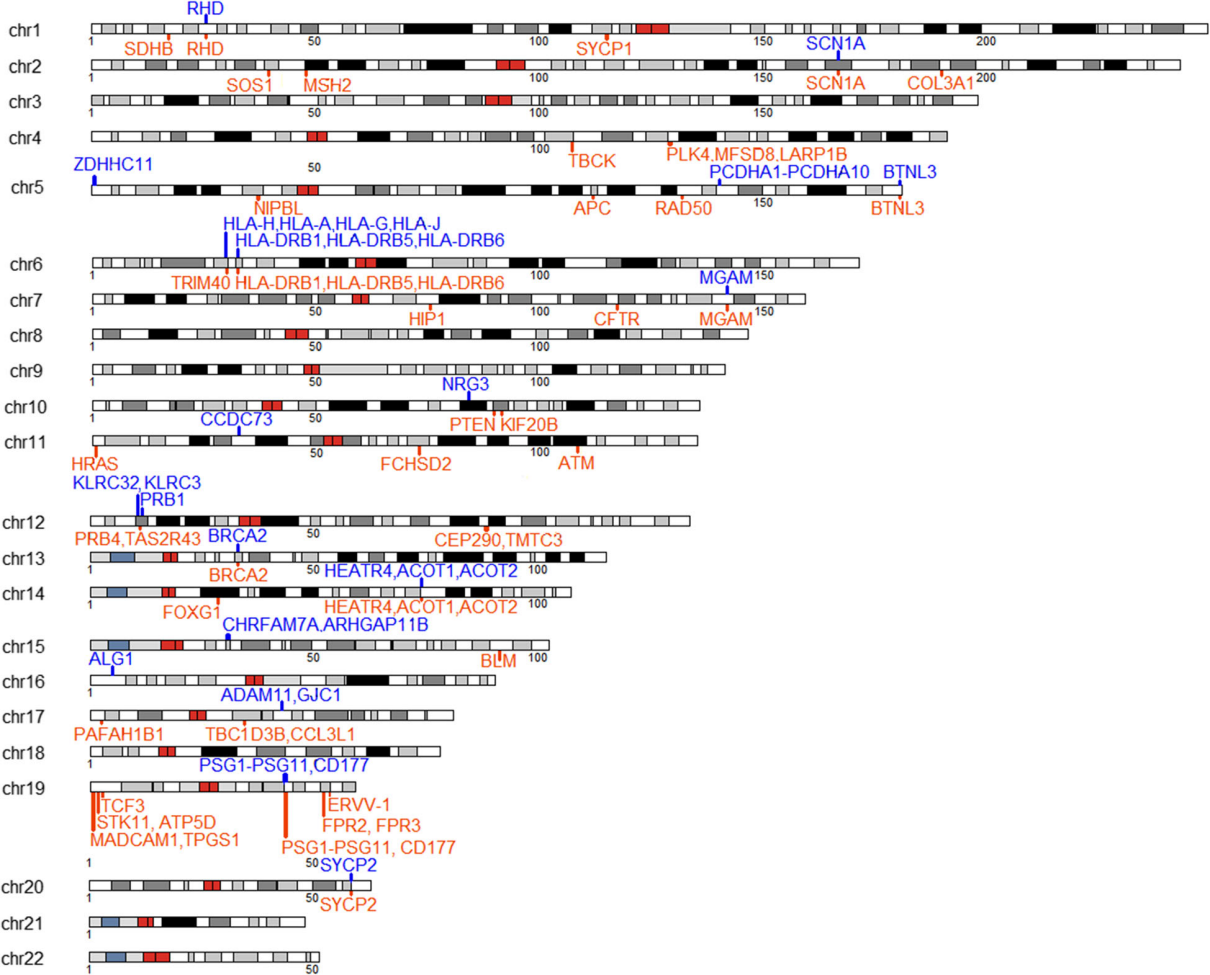
Karyoplot of the distribution of the CNVRs i*n* the GLGS-UGLI cohort. Blue- and orange-coloured bars represent loss and gain events, respectively

Subsequently, we performed the same CNVR-based association in the independent cohort of GLGS-UGLI, where we identified 76 genes in 58 CNVRs that reached the nominal level of statistical significance. A total of 33 CNVRs were significant after FDR correction (Table 3). A graphic representation of the CNVRs detected is shown in Fig. 3.

**Table 3:** Significant CNVRs identified in the GLGS-UGLI cohort

Chromosome	Start (bp)	End (bp)	Length (bp)	Type	***P***-value	Adjusted ***p***-value	Gene	Cases %	Controls %
1	17,361,781	17,371,377	9598	Gain	4.70e-02	2.89e-01	*SDHB*	1.71	0.458
1	25,599,085	25,655,563	56,480	Gain	8.90e-06	1.60e-04	*RHD*	11.1	20.5
1	25,599,085	25,655,563	56,480	Loss	1.87e-05	3.19e-04	*RHD*	15.8	7.94
1	115,400,087	115,545,917	145,832	Gain	8.35e-03	7.30e-02	*SYCP1*	0	1.22
2	39,250,139	39,294,016	43,879	Gain	4.60e-07	1.05e-05	*SOS1*	0.685	5.5
2	47,672,361	47,698,203	25,844	Gain	7.06e-15	6.02e-13	*MSH2*	1.37	11.9
2	166,850,678	166,866,353	15,677	Gain	2.05e-14	1.40e-12	*SCN1A*	2.23	13.6
2	166,850,678	166,866,353	15,677	Loss	4.91e-02	2.89e-01	*SCN1A*	0.685	0
2	189,850,694	189,871,691	20,999	Gain	1.74e-03	1.97e-02	*COL3A1*	0.856	3.51
4	107,133,338	107,181,616	48,280	Gain	2.78e-06	5.57e-05	*TBCK*	0	3.05
4	128,811,117	129,098,453	287,338	Gain	7.39e-05	1.14e-03	*PLK4, MFSD8, LARP1B*	1.54	5.8
5	700,560	850,514	149,956	Loss	7.00e-03	6.69e-02	*ZDHHC11*	5.99	2.75
5	36,995,722	37,046,626	50,906	Gain	1.75e-10	6.62e-09	*NIPBL*	2.4	11.5
5	112,082,255	112,116,602	34,349	Gain	3.03e-05	4.93e-04	*APC*	1.2	5.5
5	131,915,022	131,953,804	38,784	Gain	1.06e-03	1.29e-02	*RAD50*	0.856	3.66
5	140,230,509	140,237,998	7491	Loss	9.50e-05	1.35e-03	*PCDHA1, PCDHA2, PCDHA3, PCDHA4, PCDHA5, PCDHA6, PCDHA7, PCDHA8, PCDHA9, PCDHA10*	4.11	0.763
5	180,385,378	180,425,019	39,643	Gain	1.11e-03	1.31e-02	*BTNL3*	1.54	0
5	180,385,378	180,425,019	39,643	Loss	1.60e-16	1.82e-14	*BTNL3*	8.05	0
6	29,856,571	29,911,837	55,268	Loss	3.08e-02	2.14e-01	*HLA-H, HLA-A, HLA-G, HLA-J*	4.79	2.44
6	30,105,743	30,111,610	5869	Gain	8.35e-03	7.30e-02	*TRIM40*	0	1.22
6	32,450,297	32,572,251	121,956	Gain	2.24e-06	4.77e-05	*HLA-DRB1,HLA-DRB5, HLA-DRB6*	10.4	20.2
6	32,450,297	32,572,251	121,956	Loss	4.56e-12	2.22e-10	*HLA-DRB1,HLA-DRB5, HLA-DRB6*	19.3	6.41
7	75,362,387	75,371,553	9168	Gain	1.10e-04	1.54e-03	*HIP1*	2.05	0
7	117,246,728	117,251,848	5122	Gain	3.24e-02	2.17e-01	*CFTR*	0	0.916
7	141,760,798	141,798,014	37,218	Gain	5.62e-03	5.63e-02	*MGAM*	3.42	1.07
7	141,760,798	141,798,014	37,218	Loss	1.88e-02	1.46e-01	*MGAM*	13.5	9.16
10	83,945,607	83,955,217	9612	Loss	4.91e-02	2.89e-01	*NRG3*	0.685	0
10	89,653,781	89,699,055	45,276	Gain	4.26e-11	1.82e-09	*PTEN*	0.856	8.4
10	91,468,829	91,521,366	52,539	Gain	2.81e-03	3.09e-02	*KIF20B*	0.342	2.29
11	524,055	545,087	21,034	Gain	9.39e-08	2.46e-06	*HRAS*	0.514	5.5
11	32,749,279	32,774,462	25,185	Loss	1.08e-02	8.58e-02	*CCDC73*	1.03	0
11	72,723,762	72,814,435	90,675	Gain	3.24e-02	2.17e-01	*FCHSD2*	0	0.916
11	108,098,352	108,115,754	17,404	Gain	8.21e-08	2.33e-06	*ATM*	1.2	7.18
11	108,150,237	108,190,766	40,531	Gain	1.49e-09	5.10e-08	*ATM*	2.4	10.8
12	10,568,395	10,588,422	20,029	Loss	4.25e-03	4.40e-02	*KLRC32, KLRC3*	0	1.37
12	11,226,421	11,257,845	31,426	Gain	2.30e-02	1.74e-01	*PRB4, TAS2R43*	0.171	1.37
12	11,495,064	11,567,700	72,638	Loss	3.30e-04	4.32e-03	*PRB1*	5.14	1.53
12	88,221,580	88,632,786	411,208	Gain	3.84e-06	7.27e-05	*CEP290, TMTC3*	1.54	6.72
13	32,912,277	32,921,033	8758	Gain	9.57e-13	5.44e-11	*BRCA2*	4.79	17.4
13	32,912,277	32,921,033	8758	Loss	9.27e-03	7.79e-02	*BRCA2*	1.88	0.305
14	29,225,492	29,237,924	12,434	Gain	1.08e-02	8.58e-02	*FOXG1*	1.03	0
14	73,993,018	74,051,067	58,051	Gain	2.92e-07	7.11e-06	*HEATR4, ACOT1, ACOT2*	8.22	1.98
14	73,993,018	74,051,067	58,051	Loss	5.71e-55	1.95e-52	*HEATR4, ACOT1, ACOT2*	30.3	1.07
15	30,579,250	31,092,983	513,735	Loss	4.91e-02	2.89e-01	*CHRFAM7A, ARHGAP11B*	0.685	0
15	91,331,546	91,346,951	15,407	Gain	8.35e-03	7.30e-02	*BLM*	0	1.22
16	5,105,306	5,123,005	17,701	Loss	4.91e-02	2.89e-01	*ALG1*	0.685	0
17	2,568,663	2,576,056	7395	Gain	7.67e-05	1.14e-03	*PAFAH1B1*	0	2.29
17	34,436,532	34,534,847	98,317	Gain	4.03e-02	2.64e-01	*TBC1D3B, CCL3L1*	5.82	3.36
17	42,858,950	42,896,377	37,429	Loss	2.38e-09	7.37e-08	*ADAM11, GJC1*	4.45	0
19	483,488	517,315	33,829	Gain	2.45e-02	1.78e-01	*MADCAM1,TPGS1*	0.342	1.68
19	1,219,342	1,247,118	27,778	Gain	4.25e-03	4.40e-02	*STK11, ATP5D*	0	1.37
19	1,618,982	1,627,422	8442	Gain	7.06e-03	6.69e-02	*TCF3*	0.171	1.68
19	43,279,237	43,858,478	579,243	Gain	9.37e-03	7.79e-02	*PSG1, PSG6, PSG7, PSG11, PSG2, PSG4, PSG5, PSG9, PSG3, CD177*	2.23	0.458
19	43,279,237	43,858,478	579,243	Loss	2.50e-02	1.78e-01	*PSG1, PSG6, PSG7, PSG11, PSG2, PSG4, PSG5, PSG9, PSG3, CD177*	5.65	3.05
19	52,272,131	52,356,648	84,519	Gain	4.91e-02	2.89e-01	*FPR2, FPR3*	0.685	0
19	53,518,590	53,547,846	29,258	Gain	7.70e-04	9.70e-03	*ERVV-1*	4.11	1.07
20	58,426,497	58,476,841	50,346	Gain	5.18e-18	8.84e-16	*SYCP2*	1.88	15.1
20	58,426,497	58,476,841	50,346	Loss	2.47e-02	1.78e-01	*SYCP2*	2.23	0.611

A total of 29 out of 58 CNVRs were more present in the cases compared to controls. CNVRs containing the following genes *HEATR4*, *ACOT1*, and *ACOT2* (*p*-value = 5.71 × 10^− 55^), *SYCP2* (*p*-value = 5.18 x − 10^− 18^), *BTNL3* (*p*-value = 1.60 × 10^− 16^), *SCN1A* (*p*-value = 3.01 × 10^− 14^) and *BRCA2* (*p*-value = 9.57 × 10^− 13^) reached the highest level of significance.

Furthermore, we investigated whether the two independently analysed studies shared significant CNVRs overlapping the same genes. Table 4 shows the CNVRs found in common between the AGS and GLGS-UGLI study, and reports the combined *p*-values obtained by applying Fisher’s combined probability test. We identified in common between the two cohorts CNVRs that span 11 candidate genes (*APC*, *BRCA2, COL3A1, HLA-DRB1, HLA-DRB5, HLA-DRB6, MFSD8*, *NIPBL, SCN1A, SDHB*, and *ZDHHC11)*.

**Table 4:** CNVRs found in common between the AGS and GLGS-UGLI cohorts

	AGS				GLGS-UGLI				AGS + GLGS-UGLI	
Genes	Type	*P*-value	Cases %	Controls %	Type	*P*-value	Cases %	Controls %	*P*-value	Adjusted *p*-value
*SDHB*	Loss	6.22e-04	14.5	2.48	Gain	4.70e-02	1.71	0.458	3.30e-04	3.71e-04
*SCN1A*	Gain	3.02e-02	0.763	5.79	Gain	2.06e-14	2.23	13.6	4.66e-14	1.40e-13
					Loss	4.91e-02	0.685	0		
*COL3A1*	Gain	1.15e-02	0	4.96	Gain	1.74e-03	0.856	3.51	8.47e-05	1.09e-04
	Loss	3.70e-02	6.11	0.826						
*MFSD8*	Loss	5.54e-04	13	1.65	Gain	7.39e-05	1.54	5.8	2.21e-06	3.98e-06
*ZDHHC11*	Loss	3.70e-02	6.11	0.826	Loss	7.00e-03	5.99	2.75	2.40e-03	2.40e-03
*NIPBL*	Loss	7.29e-03	6.11	0	Gain	1.75e-10	2.4	11.5	3.17e-11	7.13e-11
	Loss	3.70e-02	6.11	0.826						
*APC*	Loss	5.85e-03	13	3.31	Gain	3.03e-05	1.2	5.5	4.40e-06	6.60e-06
*HLA-DRB1, HLA-DRB5, HLA-DRB6*	Loss	3.68e-02	29	17.4	Gain	2.24e-06	10.4	20.2	1.11e-16	9.99e-16
	Loss	8.61e-03	13.7	4.13	Loss	4.56e-12	19.3	6.41		
*BRCA2*	Loss	1.15e-04	37.4	15.7	Gain	9.58e-13	4.79	17.4	3.55e-15	1.60e-14
					Loss	9.27e-03	1.88	0.305		

The primary characteristics of these genes are presented in Table 5 and their possible relationship with glaucoma are described in the discussion section below.

**Table 5:** Characteristics of the genes identified in common AGS and GLGS-UGLI cohorts. The gene coordinates are reported in the GRCh37/hg19 build

Gene ID	Position	Description	Function
*APC*	chr5: 112,073,556-112,181,936	APC Regulator of WNT Signalling Pathway	The *APC* gene encodes a tumour suppressor protein that acts as an antagonist of the Wnt signalling pathway. It is also involved in other processes including cell migration, adhesion, transcriptional activation, and apoptosis.
*BRCA2*	chr13: 32,889,617-32,973,809	BRCA2 DNA repair associated	The *BRCA2* gene is involved in maintaining genomic instability promoting efficient and precise repair of double-strand breaks.
*COL3A1*	chr2: 189,839,099-189,877,472	Collagen type III alpha 1 chain	The *COL3A1* gene encodes for the chains of type III procollagen. Collagen type III have been found in the lamina cribrosa and in the trabecular meshwork.
*HLA-DRB1*	chr6: 32,546,546-32,557,613	Major histocompatibility complex, class II, DR beta 1	The human leukocyte antigen (HLA) genes regulate the immune process promoting the adaptive immune response in the vertebrates. HLAs are membrane glycoproteins that can be divided into two major types designated class I (encoded by the genes HLA-A, -B, and -C) and class II (encoded by the genes HLA-DR, -DP, and -DQ).
*HLA-DRB5*	chr6: 32,485,130-32,498,064	Major histocompatibility complex, class II, DR beta 5	
*HLA-DRB6*	chr6: 32,520,490-32,527,779	Major histocompatibility complex, class II, DR beta 6	
*MFSD8*	chr4: 128,838,960-128,887,139	Major Facilitator Superfamily Domain Containing 8	The *MFSD8* gene encodes for ubiquitous integral membrane protein that contains a transporter domain and a major facilitator superfamily domain. Other members of the major facilitator superfamily transport small solutes through chemiosmotic ion gradients. The protein likely localizes to lysosomal membranes.
*NIPBL*	chr5: 36,876,861-37,065,921	NIPBL cohesin loading factor	The *NIPBL* is involved in guaranteeing the cohesin complex in the chromatin and also between the enhancers and core promoters.
*SCN1A*	chr2: 166,845,670-166,930,180	Sodium voltage-gated channel alpha subunit 1	The SCN1A is a protein coding gene that encodes for sodium channel alpha subunits which are involved in the generation and propagation of action potential in nerve and muscle.
*SDHB*	chr1: 17,345,217-17,380,527	Succinate Dehydrogenase Complex Iron Sulfur Subunit B	The *SDHB* gene encodes for proteins that interact with the complex II of the respiratory chain, which is specifically involved in the oxidation of succinate and carries electrons from NADH to CoQ. The complex is composed of four nuclear-encoded subunits (named A to D) and is localized in the mitochondrial inner membrane.
*ZDHHC11*	chr5: 795,720-851,101	Zinc finger DHHC-type containing 11	The *ZDHHC11* is a protein coding gene member of the DHHC domain responsible for protein palmitoylation by protein acyltransferases with zinc-finger and Asp-His-His-Cys sequence (zDHHC) where its activity is required for the activation to DNA damage.

### Functional gene annotation, overrepresentation and pathway analysis

For all genes located in the relevant CNVR regions identified in the AGS cohort (Table 2), we conducted a functional classification and overrepresentation test using the PANTHER Classification System and applying the Bonferroni correction. We included 16 candidate glaucoma disease genes and we annotated their molecular function, biological process, cellular component and protein class. Functional classification of the genes showed that the most represented molecular function term was “protein binding”, “cellular component organization or biogenesis” in biological process, “plasma membrane” in cellular component, and “cytoskeletal protein” in protein class (specifically the “intermediate filament” and “defense/immunity protein”). The overrepresentation analysis showed that the most enriched cellular processes were the “hemidesmosome” (fold enrichment= > 100; *p*-value = 0.025) and “intermediate filament” (fold enrichment= > 100; *p*-value = 0.018). Complete results of function classification and overrepresentation analysis are reported in Supplementary data 1 and 2.

The same analyses were conducted independently in the GLGS-UGLI cohort where we included 76 candidate glaucoma disease genes. Functional classification of the genes showed that the most represented molecular function term was “protein binding”, “cellular metabolic process” in biological process, “plasma membrane” in cellular component, and “cell adhesion molecule” in protein class, specifically cadherin.

The overrepresentation analysis showed statistical significance in the category biological process, in which the “homophilic cell adhesion via plasma membrane adhesion molecules” was over-represented (fold enrichment = 29.04; *p*-value = 2.02 × 10^− 8^), in the category molecular function the “peptide antigen binding” (fold enrichment = 78.21; *p*-value = 3.01 × 10^− 5^). In the cellular component category, the most enriched class was the “integral component of plasma membrane” (fold enrichment = 5.56; *p*-value = 2.47 × 10^− 7^). The results of function and overrepresentation analysis are reported in Supplementary data 1 and 2.

The statistically significant 92 associated genes overlapping in CNVRs from the AGS and GLGS-UGLI cohorts (Supplementary data 1) were used to conduct a pathway analysis using the PANTHER classification system. The analyses resulted in overrepresentation of “cadherin signalling” (*p*-value = 2.23 × 10^− 8^), “p53 pathway feedback loops 2” (*p*-value = 1.21 × 10^− 2^), and “Wnt signalling” (*p*- value = 2.01 × 10^− 6^) to be overrepresented, using Bonferroni correction (Table 6).

**Table 6:** Pathway analysis using the PANTHER classification system in genes reported in the CNVRs identified in both cohorts

***PANTHER*** Pathways	Number of genes	Fold Enrichment	Direction (+/−)	***P***-value	Genes
Cadherin signalling pathway	11	16.29	+	2.23e-08	*PCDHA1, PCDHA2, PCDHA3, PCDHA4, PCDHA5, PCDHA6, PCDHA7, PCDHA8, PCDHA9, PCDHA10, TCF3*
p53 pathway feedback loops 2	4	18.96	+	1.21e-02	*PRB1, PTEN, ATM, HRAS*
Wnt signalling pathway	12	8.97	+	2.01e-06	*APC, PCDHA1, PCDHA2, PCDHA3, PCDHA4, PCDHA5, PCDHA6, PCDHA7, PCDHA8, PCDHA9, PCDHA10, TCF3*

After this step, we further tested these pathways with a one-way enrichment analysis in the cohort with a greater number of POAG cases, the GLGS-UGLI cohort. The *--cnv-enrichment-test* showed significance in the “cadherin signalling” (*p*-value = 0.0004), “p53 pathway feedback loops 2” (*p*-value = 0.0048) and “Wnt signalling” (*p*-value = 0.0008).

### Expression of candidate glaucoma disease genes located in associated CNVRs

To assess the expression of the 92 genes located in the significant CNVR identified in both cohorts (Tables 2 and 3), we examined the online Ocular Tissue Database (https://genome.uiowa.edu/otdb/) [65]. These results are reported in Table 7.

**Table 7:** Gene expression values from the Ocular Tissue Database (OTDB), indicated as Affymetrix Probe Logarithmic Intensity Error (PLIER) normalized value. In bold are reported the genes found in common in the AGS and GLGS-UGLI cohorts

		Ocular Tissues			
	Gene	Optic Nerve	Optic Nerve Head	Retina	Trabecular Meshwork
***90th percentile***	***APC***	163.958	209.972	–	–
	*FCHSD2*	–	134.694	102.657	–
	*GJC1*	–	–	–	413.055
	*HRAS*	–	–	115.939	–
	*MSH2*	–	–	104.639	–
	***NIPBL***	117.136	165.756	135.751	141.999
	*PAFAH1B1*	1328.740	1130.570	1181.810	1373.830
	*RAD50*	154.762	243.466	135.328	207.515
	*SOS1*	–	139.286	–	135.202
***10th percentile***	*ARHGAP11B*	9.146	7.245	8.961	6.504
	*ATM*	–	–	–	10.924
	***BRCA2***	12.279	10.740	13.335	9.430
	*CCDC73*	10.275	5.375	7.035	4.718
	*FPR2*	11.307	13.881	8.970	10.865
	***HLA-DRB1***	2.499	10.861	1.764	–
	***HLA-DRB5***	2.499	10.861	1.764	–
	*KIF20B*	11.902	13.712	–	–
	*KLRC3*	–	8.977	–	5.348
	*MGAM*	–	–	12.893	13.025
	*PLK4*	–	12.940	–	13.407
	*PRB1*	13.405	12.965	6.413	9.600
	*PRB4*	13.405	12.965	6.413	9.600
	*PSG1*	6.338	12.973	–	7.741
	*PSG3*	6.338	9.363	–	7.741
	*PSG4*	6.338	9.363	–	7.741
	*PSG6*	–	12.973	–	–
	*PSG9*	–	12.973	–	–
	*PTEN*	0.127	7.405	3.929	9.512
	*RHD*	5.402	–	11.212	3.473
	***SCN1A***	13.703	10.769	–	8.860
	*SYCP2*	12.574	10.169	7.668	8.315
	*TBC1D3B*	6.780	2.636	8.218	2.528

The *NIPBL*, *PAFAH1B1* and *RAD50* genes were found highly expressed in ON, ONH, retina and TM, with the highest level of expression for the *PAFAH1B1* gene. Conversely, the *ARHGAP11B*, *BRCA2*, *CCDC73*, *FPR2*, *PTEN*, *SYCP2* and *TBC1D3B* genes were found lower expressed in ON, ONH, retina and TM. In the ON, the lowest expression was for the *PTEN* gene, in the ONH and TM for the *TBC1D3B* gene, and in the retina for the *HLA-DRB1* and *HLA-DRB5* genes.

## Discussion

To date, the principal causes of POAG are not fully elucidated. Numerous studies suggest the involvement in the disease of several cells and structures (including the TM, chamber angle, cornea), capable of influencing intra-ocular aqueous flow and pressure. Differences in the viability of RCGs or the structure and function of the lamina cribrosa have been also found to be implicated. All these mechanisms underlying POAG disease have a common pathological consequence consisting in an optic neuropathy, most likely caused by the variation in difference between the IOP and the cerebrospinal fluid pressure over the optic disc [67]. We investigated whether CNVRs can help to identify new glaucoma candidate disease genes, and to detect molecular mechanisms underlying POAG. In two separate cohorts, we reported that POAG patients have a greater burden of CNVRs compared to controls, while in the association analysis we found differences in the frequency of CNVRs between cases and controls. Part of these CNVRs, identified and associated under stringent quality parameters, were in common between the two independent cohorts, and contained 11 genes: *APC, BRCA2, COL3A1, HLA-DRB1, HLA-DRB5, HLA-DRB6, MFSD8, NIPBL, SCN1A, SDHB*, and *ZDHHC11*. Functional annotation and pathway analysis of all the 92 genes suggested involvement of Wnt signalling, p53 and cadherin components related pathways. The possible involvement of these pathways in POAG are further discussed below.

In this study, there are a number of strong methodological points as well as a number of limitations. Strong points are the detection of CNVs from three CNV calling methods in order to establish high-quality CNV, stringent quality control at all levels, conservative choice of analysis parameters, independent confirmation and overlap in two cohorts with the same genetic background but different study design. Potential limitations are the lack of tests of CNVs in sex chromosomes, as well as the eventual functional impact of the CNVRs revealed in deletion and duplication state. This choice was driven by the awareness that cautions regarding the interpretation of clinical meaning of the CNVs should be taken. Indeed, the biological impact of CNVRs can depend on many factors, such as the genotype, the alleles that are present in a CNV, and the presence of the CNVR near enhancers and repressors factors [68, 69]. For these reasons, subsequent functional genetic studies are needed to corroborate the potential role of the POAG-CNVR identified in our investigation.

Some investigations have suggested direct links between Wnt signalling and glaucoma [70, 71]. One of them is the impact of β-catenin (component of the cell adhesion and transducer of extracellular signals) on gene expression; for example, the most common glaucoma-causing gene, MYOC, influences the intracellular levels of this molecule. Another known mechanism of Wnt is linked to the changes in adhesion junctions and cell contact. This may influence the aqueous humour outflow resistance [70–74]. The secretion of a Wnt pathway antagonist, which sequesters extracellular Wnt ligands, called secreted frizzled-related protein 1 (sFRP1) can increase in number in the TM of glaucomatous eyes. Moreover, when sFRP1 is added to ex vivo perfusion-cultured human eyes, the level of β-catenin declines [71, 75–77]. Interestingly, the Wnt ligands receptors and pathway regulators are not only expressed in the TM, but also localized in the ganglion cell layer. Consequently, Wnt signalling may exert an effect on different cell types involved in POAG pathology. Studies have also shown that the Wnt signalling is involved in the regeneration of the optic nerve after injury [78].

A number of previous studies indicated that the Wnt/β-catenin pathway plays a role also in the IOP regulation, a major risk factor of glaucoma. Wnt signalling is regulated through receptor mediated fluctuating levels of cytoplasmic β-catenin protein in human TM cells [79–81]. In the so called OFF-state Wnt signalling is not present, and β-catenin is phosphorylated by the Wnt/ β-catenin inhibitory complex through the scaffolding proteins of Axin and APC. These proteins form a destruction complex, which allows phosphorylated β-catenin to be recognized and degraded by the proteasome [82]. Therefore, in this status β-catenin will remain constantly degraded in the cell. In the Wnt-ON state, β-catenin moves into the nucleus due to the disassembly of the β-catenin complex, and binds to transcription factors [83].

In our study, POAG relevant CNVRs were found encompassing the *APC (*Regulator of WNT Signalling Pathway) gene that encodes a tumour suppressor protein. It is also involved in other processes including cell migration, adhesion, transcriptional activation, and apoptosis. The *APC* gene encodes for an antagonist of the Wnt signalling pathway and induces the degradation of β-catenin. As previously reported, this signalling is a critical regulator of outflow facility and IOP in the TM, providing a mechanistic role for its regulation [80, 84]. Evaluation of the expression data of the *APC* gene in the OTDB database showed that it was included in the 90th percentile in both the ON and ONH (Table 7). Since the Wnt signalling pathway is involved in the regeneration of the optic nerve, the presence of CNVs in the *APC* gene could affect the regulatory activity that this gene has on β-catenin. This could happen through the impact on the transcriptional regulations of the Wnt/β-catenin pathway in TM cells that perturbs the homeostasis of the IOP production and the possibility of regeneration of the ON. Other studies have raised the possibility that the 5q22 region might harbour common genetic factors regulating glaucoma risk since the *WDR36* gene is located in that area (5q22.1, locus GLC1G). *WDR36* is involved in 18S RNA processing and was identified as a causative gene of POAG [85] Reduction of *WDR36* mRNA in human TM cells in culture causes apoptosis and upregulation of *P53* mRNA [86]. Loss of Wdr36 functions lead to an activation of the p53 stress–response pathway, which suggest that co-inheritance of defects in p53 pathway genes might influence the impact of *WDR36* variants on POAG [87]. In the near chromosomal region 5q22.2 is located the *APC* gene, in which we identified statistically significant CNVRs. Evidence is reported for a link between β-catenin and p53 tumour suppressor, in which an increased expression of β-catenin induces an accumulation of p53. For example, in colorectal cancer this increase is an early event of carcinogenesis, followed by inactivation of the p53 [88]. This leads to the hypothesis that a complex mechanism in the 5q22 region could exert an effect in the susceptibility to glaucoma. Perhaps, through the alteration of the regulations in the Wnt pathway, as described above.

Interestingly, a gene involved in the “double-strand break repair” and the item “p53 pathway” emerged in our study. CNVs were found in the *BRCA2* gene that is involved in maintaining genomic stability promoting efficient and precise repair of double-strand breaks (DSBs), for which a functional link between *BRCA2* and p53 has been shown [89, 90]. Specifically, the *BRCA2* gene plays a role in the repair of the consequences of DSBs [91]. The accumulation of DSBs in the retinal neurons cause irreversible process of apoptosis [92, 93]. Evaluation of the expression data of *BRCA2* in POAG relevant ocular tissues showed that its expression was included in the lowest decile (Table 7). Our data together with other evidence reported in the literature may suggest that POAG patients can be more susceptible to the disease because of a higher genomic instability. We speculate that the presence of CNVs in the *BRCA2* gene could cause a higher expression, leading to higher genomic instability in DSBs repair. Obviously further investigations are essential to corroborate this hypothesis.

Other biological processes that emerged from our analysis were binding proteins and cell adhesion (hemidesmosome and cadherin), which are discussed next. Desmosomes and hemidesmosomes are cell-surface attachment sites in epithelial cells. Their function is to mediate anchorage at sites of cell-cell and cell-substrate contact [94]. RGCs axons make connections with other axons and glial cells through desmosome and hemidesmosome junctions, especially in their unmyelinated region within the eye, for the transport of metabolites [95–97]. RGCs and their axons have an exceptionally high energy demand to maintain their signalling function and are, consequently, rich in mitochondria. A reduction in oxygen supply to the ONH, due to alteration in the function of cell adhesion, causes a dysfunction in the status of these cells reducing their chance of survival and, as a consequence, may trigger apoptosis, which is also at the base of the pathophysiology of glaucoma [96, 98, 99]. This mechanism is also supported by the fact that degeneration and apoptotic death of RGCs axons is widely observed in this disease [100]. Other proteins involved in cell adhesion are the cadherins. The cytosolic domains of these transmembrane proteins bind directly to β-catenin. This molecule is included in the Wnt signalling pathway and interacts with adherens junctions. Thus, it is likely that Wnt signalling regulates the anchoring of cadherin junctions to the actin cytoskeleton by regulating the β-catenin [101]. In the TM, cell adhesion proteins determine the outflow resistance in aqueous humour. Consequently, we hypothesize that these proteins might cause an alteration of the ocular hypertension, a primary risk factor for POAG.

In our study, CNVRs were also reported in the *COL3A1, HLA-DRB1, HLA-DRB5, HLA-DRB6, MFSD8, NIPBL, SCN1A, SDHB,* and *ZDHHC11* genes.

The *COL3A1* gene encodes for the chains of type III procollagen. Collagen type III have been found in the lamina cribrosa and in the TM [102, 103]. A study that explored the expression of genes using expression profiles data from TM tissue, found a different expression of COL3A1 gene in patients with POAG compared to controls. This gene was considered together with *COL4A4*, *COL1A2*, *ITGB5*, *COL5A2*, and *COL5A1*, to be involved in the pathogenesis of POAG, because of its participation in extracellular matrix–receptor interaction and focal adhesion [104].

We also observed a potential association between glaucoma and CNVR spanning the HLA cluster genes. The human leukocyte antigen (HLA) genes regulate the immune process promoting the adaptive immune response [105]. HLAs are membrane glycoproteins that can be divided into two major types designated class I (encoded by the genes HLA-A, −B, and -C) and class II (encoded by the genes HLA-DR, −DP, and -DQ) [106, 107]. Studies have suggested that the autoimmune system could have a role in the development of glaucoma: a higher frequency of the HLA-DRB1*0407 haplotype was reported in Mexican POAG patients compared to controls [108, 109]. Therefore, HLA class II gene polymorphisms may influence the development of autoimmunity in glaucoma.

The *MFSD8* (Major Facilitator Superfamily Domain Containing 8) gene encodes for an integral lysosomal membrane protein that contains two domains: a transporter and a major facilitator superfamily. The latter transports small solutes through chemiosmotic ion gradients [110, 111]. The *MFSD8* gene is mainly expressed in the brain and the eyes, predominantly in the photoreceptor synaptic terminals in the retina. Variants in this gene were found to be associated with non-syndromic macular dystrophy [112–114]. Mutations in the *MFSD8* gene cause the neuronal ceroid lipofuscinosis 7 disease, characterized by the accumulation of proteins and other substances in lysosomes. In the juvenile form of this disease, a possible ocular complication is represented by secondary acute glaucoma [115].

The next POAG CNVR associated candidate gene in our study is the *NIPBL* gene. This gene is involved in the so called cohesin complex that mediates cohesion between sister chromatid and also facilitate the enhancer–promoter interaction [116, 117]. Interestingly, mutations in the *NIPBL* gene are responsible for Cornelia de Lange syndrome, a multisystem congenital disorder that is characterized by dysmorphic facial features, growth retardation, gastroesophageal dysfunction, cardiac, and ocular anomalies, like glaucoma [118–121]. Thus, genetic variations in this gene may influence facial and eye development and contribute to POAG pathogenesis.

We also found a potential association between POAG and the *SCN1A* gene*.* This gene is a protein coding gene that encodes for sodium channel alpha subunits (NaV 1.1) which are involved in the generation and propagation of action potential in nerve and muscle. Allelic variants of this gene are associated with epilepsy, febrile seizures and epileptic encephalopathy [122, 123]. NaV 1.1 has been shown to be expressed by RCGs [124–126]. Due to high energy demands, the unmyelinated RGCs axons are rich in voltage-gated sodium channels, which are essential for action potential initiation and regeneration [127].

In our study, we observed a potential association between POAG and CNVRs spanning the *SDHB* (Succinate Dehydrogenase Complex Iron Sulphur Subunit B). This is a protein coding gene, and its product is one of the five proteins of the SDH complex (SDHA, SDHB, SDHC, SDHD and SDHAF2). The SDH complex is localized in the mitochondrial inner membrane and interacts with the complex II of the respiratory chain that is specifically involved in the oxidation of succinate and carries also electrons to CoQ [128]. Usually the mitochondrial electron transport chain uses oxygen, when it is abundant, as the last electron acceptor. In the retina, mitochondria are instead specialized to reverse the activity of the SDH enzyme, in order to reduce the oxidative stress in this tissue [129]. In ONH, there is a high concentration of mitochondria due to its dependency on the adenosine triphosphate [130]. The characteristic event of RGC apoptosis for POAG patients, can be caused by the damage occurred to the mitochondria, and by the reactive oxygen species (ROS) produced [131]. Since the retina is continuously in a state of low oxygen saturation, the retinal mitochondria are specialized to reverse the SDH enzyme activity reducing fumarate (as terminal electron acceptor) to succinate. As a result, the neural retina transfers the locally produced succinate to a relatively high oxygen saturated tissue, the retinal pigment epithelium (RPE)-choroid complex, thereby reducing their oxidative stress [129]. Therefore, because the *SDH* gene activity is involved in both the directions of succinate production, genetic alterations of the activity of the SDH complex could have effect on the survival of the cells of retina and on the RPE-choroid complex.

The data from our study also suggest an association between POAG and CNVRs encompassing the *ZDHHC11* gene. The *ZDHHC11* is a member from a protein family containing the DHHC domain. This domain is responsible for protein palmitoylation through acyltransferase activity of zinc-finger and Asp-His-His-Cys sequence (zDHHC). The protein palmitoylation activity is required for the activation to DNA damage [132]. Interestingly, one of the proteins that is palmitoylated is called CAV-1 [133]. The *CAV1* gene has been consistently associated with POAG in GWASs, and its protein has most likely a role in regulating the IOP via modulation of aqueous humour drainage in the TM [10, 134].

It is relevant to notice that we did not detect previously reported CNV associations with POAG, e.g. those reported in the gene *GALC*, due to the specific approach used in this study. Here we aimed to identify molecular mechanisms that underlie POAG through the investigation of CNVs that were common (a frequency of more than 1% in each of our cohort). Conversely, the previous studies have been focused on the research of rare CNVs associated with POAG [34, 35, 135, 136].

From our investigation of the gene expression levels in ON, ONH, retina and TM, the highest level of expression was found for the *PAFAH1B1* gene, also known as LS1. This gene encodes the non-catalytic alpha subunit of the intracellular Ib isoform of platelet-activating factor acetylhydrolase complex. The complex is an enzyme composed of three subunits encoded by the *PAFAH1B1*(the regulatory subunit), *PAFAH1B2* and *PAFAH1B3* genes [137]. Diseases associated with *PAFAH1B1* include Lissencephaly 1 and Miller-Dieker Lissencephaly Syndrome [138]. A study by Nabi et al. reported that of 20 patients with lissencephaly, 19 of them had ocular abnormalities, including optic nerve hypoplasia and atrophy, retinal dysplasia, and retinal nonattachment [138, 139]. Recent studies revealed that *PAFAH1B1* is involved in the dynein/dynactin-motor complex (the retrograde transport from axons to cell body), and that the increased accumulation of dynein in the ONH and retina causes elevated IOP [140, 141]. Moreover, the *PAFAH1B1* gene interacts with *DAB1,* a transducer of the reelin signal that plays a role in the ON-OFF organization and attenuation of rod-driven retinal responses [142–145].

On the other hand, in our study the *TBC1D3B* gene showed the lowest level of expression in the ON, ONH, retina and TM. The TBC1D3B protein contains a TBC domain involved in the RAB GTPase signalling, by activating protein for *RAB5* [146]. Rab5 participate in the fast retrograde transport responsible for removing damaged or misfolded proteins from the ON to the RGCs [147, 148]. Mencarelli and co-authors have identified a duplication of 1.8 Mb in 17q12, including the *TBC1D3* gene in a patient with Peters’ anomaly, microphthalmia, and glaucoma [149]. We speculate that a CNV might destabilize the functionality of this gene leading to an inactivation of the RAB GTPase signalling. As a consequence, this could cause an inhibition of the fast retrograde transport of axonal vesicular trafficking system [150]. These events may make the RGCs more vulnerable to damage, thus leading to the manifestation of glaucoma due to the lack of transmission factors (like the brain-derived neurotrophic one) that contribute to cell death [151].

## Conclusion

In conclusion, we reported that POAG patients have a greater burden of CNV compared to controls, and that the CNVs most likely affect genes that belong to cell adhesion components, Wnt and p53 pathways. Our data combined with those of the literature suggest that CNVs may have a role in the susceptibility of POAG, and that they can reveal more information on the mechanism behind this disease. Since data from CNV studies are still scarce, more genetic commitment is warranted to ascertain the contribution of CNVs in POAG.

## Supplementary Information

**Additional file 1.**

## Data Availability

The data that support the findings of this study are available from Lifelines biobank but restrictions apply to the availability of these data, which were used under license for the current study, and so are not publicly available. Data are however available from the authors upon reasonable request and with permission of Lifelines.
